# Hospitalized COVID-19 Patients with Urinary Tract Infection in Iran: *Candida* Species Distribution and Antifungal Susceptibility Patterns

**DOI:** 10.3390/antibiotics13070633

**Published:** 2024-07-08

**Authors:** Zeinab Soleimani Shiyadeh, Shirin Farahyar, Laleh Vahedi Larijani, Justin Beardsley, Noura Nouri, Shahram Mahmoudi, Shahla Roudbar Mohammadi, Célia Fortuna Rodrigues, Maryam Roudbary

**Affiliations:** 1 Department of Parasitology and Mycology, School of Medicine, Iran University of Medical Sciences, Tehran 1449614535, Iran; susan.soleymani23@gmail.com (Z.S.S.); sh_farahyar@yahoo.com (S.F.); sh.mahmoudi93@gmail.com (S.M.); 2 Microbial Biotechnology Research Center (MBiRC), School of Medicine, Iran University of Medical Sciences, Tehran 1449614535, Iran; 3 Department of Pathology, School of Medicine, Mazandaran University of Medical Sciences, Sari 48175-866, Iran; lalevahedi@gmail.com; 4 Sydney Infectious Diseases Institute, University of Sydney, Sydney, NSW 2145, Australia; justin.beardsley@sydney.edu.au; 5 Westmead Hospital, NSW Health, Sydney, NSW 2145, Australia; 6 Department of Mycology, Faculty of Medical Sciences, Tarbiat Modares University, Tehran 14115111, Iran; nouranouri932@gmail.com (N.N.); sh.mohammadi@modares.ac.ir (S.R.M.); 7 Associate Laboratory i4HB—Institute for Health and Bioeconomy, University Institute of Health Sciences—CESPU, 4585-116 Gandra, Portugal; 8 UCIBIO—Applied Molecular Biosciences Unit, Translational Toxicology Research Laboratory, University Institute of Health Sciences (1H-TOXRUN, IUCS-CESPU), 4585-116 Gandra, Portugal; 9 LEPABE—Laboratory for Process Engineering, Environment, Biotechnology and Energy, Faculty of Engineering, University of Porto, 4200-465 Porto, Portugal; 10 ALiCE—Associate Laboratory in Chemical Engineering, Faculty of Engineering, University of Porto, 4200-465 Porto, Portugal

**Keywords:** *Candida* species, candiduria, COVID-19, antifungal susceptibility

## Abstract

*Candida* species, typically part of the human skin and mucous membrane flora, can cause opportunistic fungal infections, notably urinary tract infections (UTIs), which are on the rise among hospitalized COVID-19 patients. The lack of understanding of UTIs in this population, coupled with the emergence of multidrug-resistant strains, poses significant challenges for effective treatment and further investigations. In this study, urine samples were collected from 70 COVID-19 patients with UTIs in sterile containers for microbiology examination. After microscopic observation, the isolates were identified both by phenotypic and molecular techniques such as multiplex PCR. Antifungal susceptibility testing (AFST) against fluconazole (Flu), itraconazole (Itr), and amphotericin B (AMB) was performed according to CLSI M27/S4 standard methods, with the frequency of isolates including *Candida albicans* (*n* = 20, 51.3%), *Candida tropicalis* (*n* = 15, 38.4%), *Nakaseomyces glabrata* (previously *Candida glabrata*) (*n* = 2, 5.1%), *Pichia kudriavzevii* (previously *Candida krusei)*, and *Candida parapsilosis* (*n* = 1, 2.5%). All isolates of *C. albicans*, *C. tropicalis*, *C. glabrata*, and *C. parapsilosis* were sensitive to amphotericin B, while *C. kruzei* was resistant to AMB. Around 70% of *C. albicans* isolates were sensitive to Flu; 20% of *C. tropicalis* were resistant to itraconazole, while 33% were resistant to fluconazole. *C. albicans* and *C. tropicalis* were the main causes of candiduria in infected cases and both Flu and AMB showed good results in AFST in these species. Performing drug susceptibility testing for clinical isolates of *Candida* spp. provided guidance for appropriate management and control, and timely antifungal treatment.

## 1. Introduction

*Candida* species can colonize skin and mucosal surfaces and, given the right conditions, can cause opportunistic infections—the most worrying of these are blood stream infections but many other body sites can be affected, including the genitourinary system [1]. Coronavirus disease 2019 (COVID-19) has become a global issue because of its high prevalence leading to hundreds of millions of deaths and affecting healthcare systems worldwide. It has been clearly demonstrated that the incidence of opportunistic fungal infections increased in relation to the COVID-19 pandemic. Overall, rates in hospitalized patients especially ICU-admitted cases (9.6%) were significantly greater during the COVID-19 pandemic than prior [2,3,4].

A wide range of bacteria, viruses, and fungi can cause UTI. Although the majority are caused by bacteria, around 10–15% are caused by *Candida* species [5]. Candiduria—the presence of *Candida* species in urine—is common in at-risk inpatients such as those with diabetes mellitus, long-term use of antibiotics and corticosteroids, hospitalized people with a urinary catheter, as well as critically ill patients like those with COVID-19 [6]. COVID-19 patients can experience immunosuppression, particularly due to the use of steroids, attributing it to dysregulated immune responses. This creates a favourable environment for the development of persistent fungal co-infections. Additionally, the collateral effects of host recognition pathways, crucial for activating antiviral immunity, induce a highly permissive inflammatory environment, promote fungal pathogenesis, and increase the susceptibility of COVID-19 patients to opportunistic fungal infections [2]. Candiduria is defined as 10^4^–10^5^ CFU/mL of yeasts detected from urine, whereas a *Candida* UTI is mainly characterized by 10^5^< CFU/mL being detected, which varies from asymptomatic to some clinical presentation of UTI include dysuria, frequent and suprapubic pain as well as puria to invasive candidiasis [7,8]. Although therapy is not necessitated in colonized patients [9], effective and timely treatment is recommended in cases with predisposing factors and patients with clinical signs of infection [10]. Candiduria can signal systemic candidiasis, including candidemia, though this is rare from UTIs alone. In immunocompromised individuals or those with underlying medical conditions, *Candida* in urine may lead to bloodstream infection. Thus, systemic candidiasis should be considered with candiduria, particularly in high-risk patients. It has been reported that in critically ill patients or those undergoing surgical procedures, candidemia rates from UTI sources may reach up to 10% [11].

The diversity of *Candida* species in candiduria patients has been reported from various studies. Although *C. albicans* is still the predominant agent in UTI, non-*albicans Candida* (NAC) such as *C. glabrata*, *C. krusei*, *C.parapsilosis*, *C. tropicalis*, *Candida kefyr*, *Candida dubliniensis*, or *Candida guilliermondii* (*Meyerozyma guilliermondii*), has increased over the world in clinical settings [12]. Recently, *Candida auris* was also isolated from a candidemia patient secondary to chronic UTI [13]. Unfortunately, several of these species have intrinsically reduced the susceptibility to antifungal drugs or the propensity to acquire resistance. This has led to a high rate of morbidity and mortality and growing concerns as it poses a significant threat to public health [14,15]. In fact, species of the genera *Candida* are among the most important yeasts in terms of pathogenicity, which have the ability to form biofilms (adherent microbial community) on multiple medical surfaces. These biofilms can form in urinary catheters, which lead to persistent *Candida* infections and higher rates of therapeutic failure [16,17]. Although some studies have addressed the most common fungal infections in COVID-19 cases in Iran [18] and other countries, including aspergillosis [19,20], mucormycosis [21,22], and candidiasis [23,24] in COVID-19 patients, candiduria’s impact on COVID-19 patients is poorly understood and data regarding *Candida* UTI are insufficient. Additionally, the epidemiology of *Candida* UTIs varies profoundly by region [25,26], and, as a consequence, the assessment of local data is vital to elucidate trends over time. Moreover, understanding the causative agents and antifungal susceptibility pattern of *Candida* spp. in COVID-19 patients with candiduria is essential for the control, optimal management, and effective treatment of disease. To address this gap, in this study, we investigated the distribution of *Candida* species isolated from hospitalized COVID-19 patients suffering from UTI in Iran with determined antifungal susceptibility testing, to gain an insight into the therapeutic options to increase the patient outcome, using appropriate antifungal treatment.

## 2. Results

### 2.1. Distribution of Candida spp. and Patient Features

In this study, 39 *Candida* species were recovered from 70 urine sample using both morphological and molecular methods. Direct examination of urine specimens indicated yeast budding cells in candiduria positive cases. C. albicans was the predominant isolate from UTI (*n* = 20, 51.3%), followed by *C. tropicalis* (*n* = 15, 38.4%), *C. glabrata* (*n* = 2, 5.1%), *C. krusei*, and *C. parapsilosis* (*n* = 1, 2.5%). The multiplex PCR result confirmed CHROMagar *Candida* identification. Patients’ characteristics are summarized in Table 1. In this study, most UTI cases were female (29, 74.35%) with fewer men (10, 25.64%). The highest rate of infection was detected among individuals aged over 60 years (51.21%), followed by those in the 30–60 age range. Diabetes type 2 was the most frequent predisposing factor in patients with UTI (46.51%). Other underlying diseases contributed to UTI detected in patients who used antibiotics and immunosuppressive drugs, and to their duration of hospital stay. None of the patients included in our study had received antifungal therapy prior to the diagnosis of UTI. The majority of patients had chronic kidney disease, or chronic obstructive pulmonary disease (COPD), were more susceptible to severe COVID-19 infections, and necessitated the use of immunosuppressive drugs for management. The length of immunosuppressive therapy depended on the patient response; generally, in our study, patients received immunosuppressive drugs for 10–14 days during hospitalization. This timeframe was based on clinical assessment and the patient’s response to therapy.

### 2.2. Antifungal Susceptibility Testing (AFST)

AFST against FLZ, ITZ, and AMB was performed according to the CLSI protocol for *Candida* spp. The result of AFST indicated that AMB (0.25 μg/mL) effectively inhibited 100% growth in all tested Candida species. ITZ (0.5 μg/mL) and FLZ (2 μg/mL) exhibited a 50% reduction in growth inhibition of Candida species isolated from patients. All isolates of *C. albicans*, *C. tropicalis*, *C. glabrata*, and *C. parapsilosis* were sensitive to AMB, except *C. kruzei*, which demonstrated resistance. Notably, 70% of *C. albicans* isolates were sensitive to FLZ and only one was detected as resistant. A total of 20% of *C. tropicalis* were resistant to ITZ and 33% were resistant to FLZ. Additionally, two isolates of *C. glabrata* were resistant to both FLZ and ITZ. One isolate of *C. krusei* was resistant to all antifungal drugs, FLZ, ITZ, and AMB, whereas *C. parapsilosis* was dose-dependently sensitive to FLU and sensitive to ITZ. AFST findings against all isolates are shown in Table 2.

## 3. Discussion

COVID-19 patients are known to be at high-risk of disseminated fungal infections, with high mortality and morbidity [27]. Nonetheless, UTIs have not been well-studied in this population. Till now, only limited data are available regarding *Candida* UTI in the COVID-19 population [28,29]. Understanding the causative agents responsible for candiduria in hospitalized COVID-19 patients, as well as the antifungal susceptibility patterns of *Candida* spp. isolates, provides valuable insights for early diagnosis, treatment strategies, and clinical outcome. UTI in critically ill and immunosuppressed patients admitted to ICU is a matter for concern in a healthcare setting due to the indication of invasive candidiasis (IC) and need for appropriate antifungal medications [30]. The mortality rate attributed to candidemia and invasive infections is approximately 30%. Concerns about fluconazole resistance primarily involve *Nakaseomyces glabrata*, *Candida parapsilosis*, and *Candida auris*, whereas *Candida* tropicalis infections exhibit less susceptibility [31]. In this study, we aimed to identify *Candida* spp. and risk factors, and to determine AFST patterns of isolates from the urine samples of COVID-19 hospitalized patients suffered from UTIs for the first time in Iran during the pandemic. The patients had encountered moderate to severe cases of COVID-19, necessitating hospitalization in the ICU and treatment with immunosuppressive drugs to manage the disease. The European Centre for Disease Prevention and Control (ECDC) recently reported *Candida* spp. as the seventh most frequently isolated microorganism causing UTI in Europe with a significant increase among critically ill patients [32].

Several studies have shown that the distribution of *Candida* spp. recovered from candiduria is changing worldwide [33,34,35], with an increasing frequency of drug-resistant NACs (e.g., *C. glabrata*, *C. tropicalis*, *C. krusei*). In our study, *C. albicans* was the most frequent *Candida* species isolated from UTI patients, followed by *C. tropicalis*, *C. glabrata*, *C. krusei*, and *C. parapsilosis*. Consistent with our findings, several studies indicated the superiority of *C. albicans* UTI over NCAs, with a rapid emergence of NACs [7,8,36,37] worldwide. In contrast, in a study by Santana in Brazil, *C. tropicalis* was found most commonly in UTI, whereas *C. albicans* was the second most common species [38], which might be explained by the different population and geographic area of study. In another recent report, from critically ill COVID-19 hospitalized patients in Brazil [29], candiduria was directly related to *C. albicans*, *C. glabrata*, and *C. tropicalis*. In our study, it was also evident that a longer hospitalization poses a significant predisposing factor with UTI. The prolonged exposure to the hospital environment may compromise the host’s immune defences, rendering them more susceptible to colonization and infection by *Candida* spp. [39]. This is corroborated by a previous study by Singulani and colleagues [29]. A similar study evaluated risk factors in hospitalized COVID-19 cases with candidiasis. *C. albicans* was the most prevalent species associated with candiduria symptoms [28].

The associated underlying diseases are similar to studies previously conducted in patients hospitalized for COVID-19 or other causes. For instance, in our study, as expected, many of the patients with indwelling urinary catheter usage showed UTIs, mirroring findings reported by Yazdanpanah [28], as catheterization and admission to the ICU is considered as an elevated risk of UTI. In our study, patients received empirical antibiotic treatment during hospitalization, and it impacted on *Candida* UTI. In studies on COVID-19 patients, 70–90% of patients received antimicrobial therapy, and only 10% had fungal or bacterial infections [40,41]. It has been investigated that long-term use of broad-spectrum antibiotics used in COVID-19 patients is a significant risk factor for fungal infection [42]. In addition, in our study, diabetes mellitus cases (a high-risk group caused by *C. albicans* and non-*Candida albicans* species [36,43,44,45]) accounted for the main group of COVID-19 patients (46.51%), further increasing the risk to UTI in our study.

Indeed, the global health burden of both diabetes and *Candida* spp. infections is on the rise, and COVID-19 and diabetes can have a compounding effect on the emerging of a fungal infection [46].

Although the general patterns of AFST against clinical isolates of *Candida* spp. have been studied, there is a lack of sufficient data associated with AFST of *Candida* isolates from COVID-19 patients with candiduria. According to our AFST findings, the majority of *C. albicans* isolates (70%) were sensitive to FLZ. FLZ is the treatment of choice for UTI due to high tissue and urine concentrations, and infrequent adverse effects [30]. None of the patients in this study received FLZ prophylaxis during the study, and no previous exposure to FLZ can account for the observed high rate of sensitivity among *Candida* spp. [47]. In the end, we can see a low rate of resistance to azole drugs among the *Candida* species recovered from UTI in our study. Additionally, two isolates of *C. glabrata* and one isolate of *C. krusei* were resistant to both FLZ and ITZ, which was also described before in some reports from Iran [28,48,49,50,51,52].

In our study, the most effective antifungals agents were FLZ and AMB, with low MIC values for *C. albicans*, a predominant isolate, in both drugs. From the perspective of availability, cost-effectiveness, and bioavailability, FLZ is a suitable drug as a first choice to define the most successful approach to the management of UTI [30]. Still, antifungal susceptibility testing is highly recommended for clinical isolates of *Candida* spp. because *C. krusei* has intrinsic resistance to FLZ [53], and *C. glabrata* species have been reported as having high MICs values or being FLZ resistant [47]. Despite there being no resistant *Candida* spp. to AMB in our study, the high cost and impracticality of AMB have led to its limited prescription in Iran for treating COVID-19 patients. As a limitation of this study, we were unable to test a variety of antifungal drugs against *Candida* spp. due to budget limitations in accessing those antifungals for this research. In addition, the number of patients was decreased due to the control of COVID-19 through vaccinations, which contributed to lowering the number of co-infections. In fact, while the sample size of this study is limited, it is reflective of the prevalence of the condition in our specific clinical setting (Emam Khomeini University hospital, Sari, Iran). Moreover, case series are often invaluable in highlighting patterns and generating hypotheses for larger studies. To fight this limitation, we ensured rigorous data collection and analysis to maximize the robustness of our findings despite the smaller cohort.

In summary, the growing knowledge of clinicians, and researchers regarding candiduria, its causative agent identification, and antifungal susceptibility pattern in high-risk patients with underlying disease, play a substantial role in the prophylaxis and effective treatments, as well as precise selection of antifungal drugs to mitigate drug resistance among *Candida* species, which has been rapidly growing these last years.

## 4. Materials and Methods

### 4.1. Patients and Clinical Specimen Processing

Urine samples were collected in a sterile container from COVID-19 patients with suspected UTI infection hospitalized in the ICU ward of Emam Khomeini University hospital, Sari, Iran, from January 2021 to September 2021. All patients had indwelling urinary catheters. This study was reviewed by ethical committee members of the Iran University of Medical sciences, and ethical approval was granted with number IR.IUMS.1400.586. The samples were transferred to microbiology laboratory immediately for microbiology assessment. Demographic data from patients (e.g., age, sex, the history of antifungal drugs and underlying disease) were recorded in the questionary form. COVID-19 was diagnosed based on positive real-time polymerase chain reaction (RT-PCR) tests for SARS-CoV-2, and candiduria was identified by *Candida*-positive urine cultures. This was a non-interventional study, and the authors did not have any influence on the prescription of antifungal treatment.

### 4.2. Identification of Isolates

#### 4.2.1. Conventional Methods and Culture Condition

All urine samples were centrifuged, and the sediment used for microscopic evaluation to observe yeast and pseudohyphae. For the microscopic observation, the urine sediment was stained using lactophenol cotton blue to highlight cell structures and detect the differentiation of yeast and pseudohyphae. At first, *Candida* species were identified by the conventional method. For this, the sediments were cultured on sabouraud dextrose agar (SDA; Merck, Darmstadt, Germany) and incubated in 37c for 48 h. *Candida* species were presumptively identified by streaking to CHROMagar *Candida* (CHROMagar™, Sigma-Aldrich, St. Louis, MO, USA) based on the production of a specific colour [54]. Additionally, yeast colonies were cultured on cornmeal agar medium (Micromedia, Budapest, Hungary) supplemented with Tween^®^ 80 (Sigma-Aldrich, USA) for the identification of *Candida* species based on their morphology.

#### 4.2.2. Molecular Assay

Molecular identification was applied to confirm *Candida* species using 21-plex PCR and specific primers for each species [55]. For this purpose, DNA was extracted from *Candida* species (fresh colony from 24 h culture) using a Pouya gene DNA extraction Kit (Tehran, Iran). The quality and quantity of DNA was checked by a NanoDrop Spectrophotometer (Thermo Fisher Scientific, Waltham, MA, USA) and gel electrophoresis, respectively. DNA was amplified in a PCR reaction. Species-specific primers [55] were used for the precise identification of each *Candida* species. Species with failed identification in the first multiplex PCR were further tested by the second multiplex PCR, and in the case of obtaining negative results, isolates were checked by a third multiplex PCR [55]. PCR products were visualized on 2% agarose gel electrophoresis and checked visually by Gel Doc (Gel Doc XR+, Bio-Rad, Hercules, CA, USA). The identification of *Candida* species was performed by comparison of the sizes of the fragments with the references’ band profiles for each species.

### 4.3. Antifungal Susceptibility Testing

All isolates were tested for susceptibility against fluconazole (FLZ), amphotericin B (AMB), and itraconazole (ITZ) by the microdilution broth method, and according to the method of the Clinical and Laboratory Standards Institute (CLSI M27-A3/S4) guidelines [56]. Briefly, dilutions were prepared in RPMI-1640 medium (Roswell Park Memorial Institute; Sigma Chemical Co., St. Louis, MO, USA) in 96-well flat-bottom microtiter plates (NuncTM, Thermo Fisher Scientific, Illkirch-Graffenstaden, France). For ITZ, (ITZ; MIC range: 0.125–16 μg/mL; Sigma-Aldrich) and AMB, concentrations ranged from 0.016 to 16 g/mL, whereas for FLZ, it ranged from 0.063 to 64 g/mL. Each *Candida* isolate was inoculated at a concentration of 0.5–2.5 × 10^3^ CFU/mL and incubated at 35 C for 24 h. *Candida parapsilosis* (ATCC 22019) and *C. krusei* (ATCC 6258) were used for quality control. The growth of fungi in the wells was checked visually. MIC50 for FLZ and ITZ was considered as the minimum concentration of drugs inhibiting 50% of fungal growth, while for AMB, the MIC endpoint was the lowest concentration that inhibited 100% of fungal growth as compared with that of the drug-free control. MIC values of FLZ and ITZ were interpreted based on clinical breakpoints (CBP), whereas MIC for AMB was considered the epidemiological cut off, as a clinical breakpoint has not been reported for AMB in CLSI M27/S4 [56].

### 4.4. Statistical Analysis

Data analysis was conducted using SPSS version 24 (IBM Corp., New York, NY, USA). All AFST experiments were carried out in triplicate, and appropriate positive and negative controls were used for tests.

## Figures and Tables

**Table 1:** Patients’ demographic data collected from medical records.

Variable		*n* (%)
Gender	Male	10 (25.64%)
	Female	29 (74.35%)
Age (20–70 years)	<30	2 (5.12%)
	30–60	17 (43.58%)
	>60	20 (51.28%)
Predisposing factors	Diabetes (type 2)	18 (46.51%)
	Immunosuppressive drugs	12 (30.76%)
	Cancer	4 (10.25%)
	Antibiotics therapy (2 weeks)	15 (38.5%)
	Length of hospitalization (6–12 days)	25 (64%)

**Table 2:** Antifungal susceptibility testing of three antifungal drugs against Candida isolates according to (CLSIM27/S4) guidelines.

*Candida* spp.	FLZ (μg/mL)			ITZ (μg/mL)			AMB (μg/mL)		
*C. albicans* (*n* = 20)	S ≤2	R ≥8	SDD 4–8	S ≤0.125	R ≥1	SDD 0.25–0.5	S ≤2	R >2	SDD --
	14 (70%)	1 (5%)	5 (15%)	5 (15%)	--	15 (75%)	20 (100%)	--	--
*C. tropicalis* (*n* = 15)	7 (47%)	3 (20%)	5 (33%)	2 (13%)	3 (20%)	10 (66%)	15 (100%)	--	--
*C. glabrata* (*n* = 2)	S	R ≥64	SDD ≤32	S ≤0.125	R ≥2	SDD 0.25–0.5	S ≤1	R ≥2	--
	--	2 (100%)	--	--	1 (50%)	1 (50%)	2 (10%)	--	--
*C. parapsilosis* (*n* = 1)	--	--	1 (100%)	1 (10%)	--	--	1 (10%)	--	--
*C. krusei* (*n* = 1)	--	1 (100%)	--	--	1 (10%)	--	--	1 (100%)	--

Note: S—sensitive; R—Resistance; SDD—susceptible dose dependent.

## Data Availability

Data are contained within the article and Supplementary Materials.

## Supplementary Materials

The following supporting information can be downloaded at: https://www.mdpi.com/article/10.3390/antibiotics13070633/s1, PCR product of *Candida* spp. recovered from urine culture of UTI patients and one figure of CHROMagar *Candida* used to the preliminary identification of *Candida* spp. based on the color production by each isolate.
