# Supplementary material for: Hospitalized COVID-19 Patients with Urinary Tract Infection in Iran: Candida Species Distribution and Antifungal Susceptibility Patterns
Source: Antibiotics (Basel). 2024 Jul 8;13(7):633. doi: 10.3390/antibiotics13070633 (PMC11273823; doi:10.3390/antibiotics13070633)
Supplement: Supplementary file 1 [file antibiotics-13-00633-s001.zip › antibiotics-3018766-supplementary.pptx]

## Slide 1
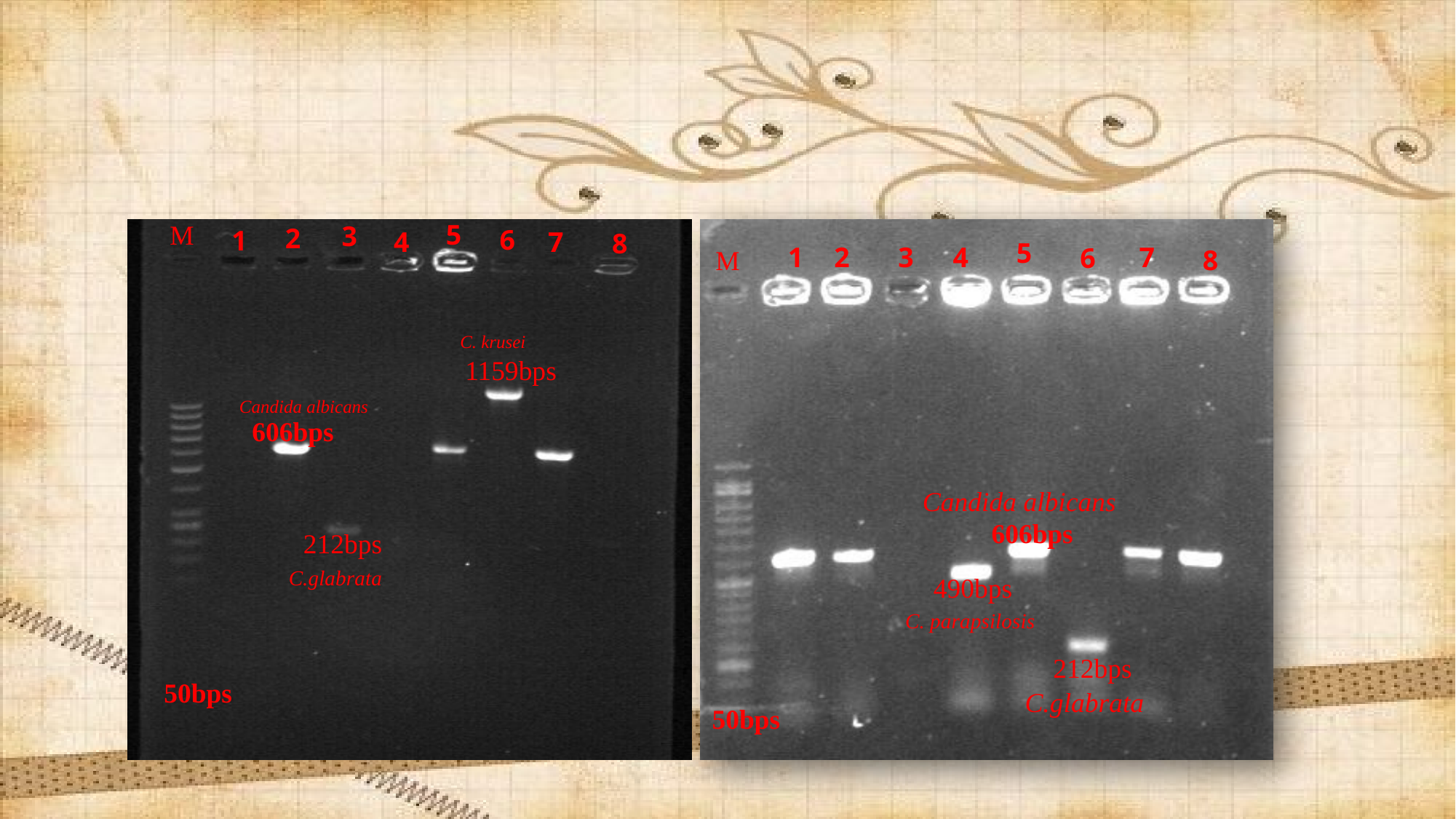

#
M
5
3
2
6
1
4
7
8
5
1
2
3
4
7
6
M
8
C. krusei
1159bps
Candida albicans
606bps
Candida albicans
606bps
212bps
C.glabrata
490bps
C. parapsilosis
212bps
50bps
C.glabrata
50bps
53

## Slide 2
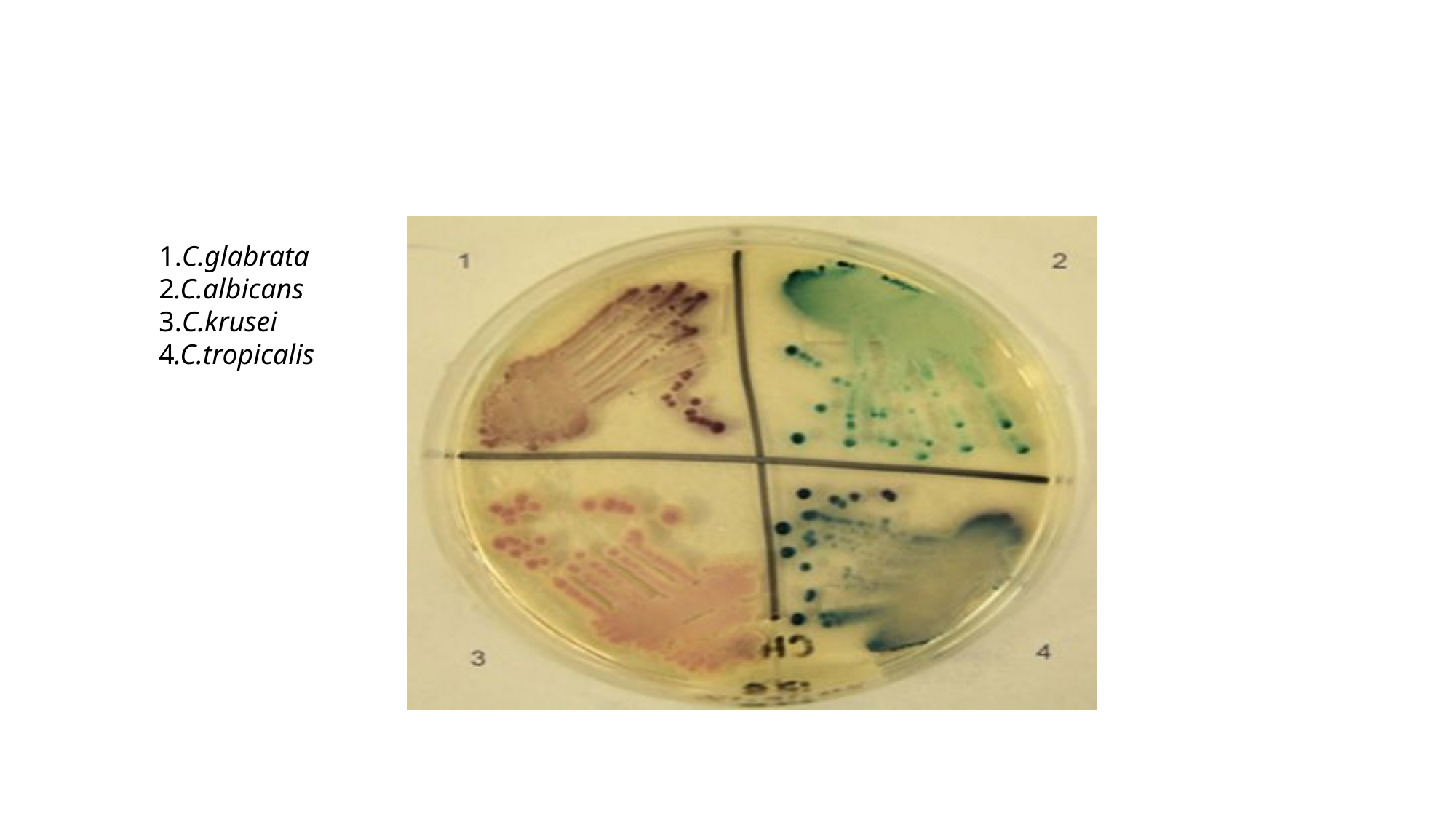

1.C.glabrata
2.C.albicans
3.C.krusei
4.C.tropicalis
